# NAA10 gene related Ogden syndrome with obstructive hypertrophic cardiomyopathy: A rare case report

**DOI:** 10.1097/MD.0000000000036034

**Published:** 2024-02-09

**Authors:** Feihong Li, Wenyang Wang, Yazhou Li, Xiwang Liu, Zhirui Zhu, Jian Tang, Yaoqin Hu

**Affiliations:** a Department of Anesthesiology, Children’s Hospital, Zhejiang University School of Medicine, Hangzhou, China; b Department of Clinical Laboratory, Huadong Hospital, Fudan University, Shanghai, China; c Department of Cardiac Surgery, Children’s Hospital, Zhejiang University School of Medicine, Hangzhou, China.

**Keywords:** case report, NAA10 gene, obstructive hypertrophic cardiomyopathy, Ogden syndrome, X-linked disorder

## Abstract

**Rationale:**

Ogden syndrome is an exceptionally rare X-linked disease caused by mutations in the NAA10 gene. Reported cases of this syndrome are approximately 20 children and are associated with facial dysmorphism, growth delay, developmental disorders, congenital heart disease, and arrhythmia.

**Patient concerns:**

We present the clinical profile of a 3-year-old girl with Ogden syndrome carrying a de novo NAA10 variant [NM_003491:c.247C>T, p.(Arg83Cys)]. During infancy, she exhibited features such as left ventricular hypertrophy, protruding eyeballs, and facial deformities.

**Diagnosis:**

Clinical diagnosis included Ogden syndrome, congenital heart disease (obstructive hypertrophic cardiomyopathy, left ventricular outflow tract obstruction, mitral valve disease, tricuspid valve regurgitation), tonsillar and adenoidal hypertrophy, and speech and language delay.

**Interventions:**

The girl was considered to have hypertrophic cardiomyopathy (HCM) and received oral metoprolol as a treatment for HCM at our hospital. The drug treatment effect was not ideal, and her hypertrophy myocardial symptoms were aggravated and she had to be hospitalized for surgery.

**Outcomes:**

The girl underwent a modified Morrow procedure under cardiopulmonary bypass and experienced a favorable postoperative recovery. No pulmonary infections or significant complications were observed during this period. The patient’s family expressed satisfaction with the treatment process.

**Lessons:**

The case emphasizes the HCM of Odgen syndrome, and early surgery should be performed if drug treatment is ineffective.

## 1. Introduction

Ogden syndrome is an extremely rare X-linked recessive genetic disorder caused by heterozygous mutations in the NAA10 gene, which encodes N-α-acetyltransferase 10, a protein involved in protein biosynthesis.^[1,2]^ Molecular studies in patients with Ogden syndrome have shown reduced N-terminal acetyltransferase protein complex A (NatA) activity and decreased N-terminal acetylation of NatA substrates. NAA10 encodes the catalytic subunit of the NatA, which is responsible for acetylating 40% to 50% of all human proteins.^[2–4]^ As a result, Ogden syndrome falls under the broader category of NAA10-related syndromes.

Since its initial discovery in 2011, just over 20 confirmed cases of Ogden syndrome have been reported. These cases exhibit distinct and recognizable phenotypes, including postnatal growth retardation, severe global developmental delay, distinctive craniofacial features, and structural heart abnormalities or arrhythmias.^[1,5]^ While the association between Ogden syndrome and obstructive hypertrophic cardiomyopathy (HCM) is rare, it has been documented. Early diagnosis is crucial, and timely surgical intervention is necessary to mitigate the impact on growth and development and improve the child’s survival time and quality of life. This case report expands our understanding of the phenotypic manifestations associated with Ogden syndrome and highlights the importance of surgical management in these cases.

## 2. Case presentation

Three years ago, 3-months-old girl with a grade 1/6 systolic murmur at the left sternal edge, between the 2nd and 3rd ribs, were taken to the hospital to our hospital. The echocardiogram (June 22, 2020) revealed an interventricular septum thickness of 8.8mm, posterior wall thickness of 5.6 mm, thickening and limited motion of the anterior leaflet of the mitral valve with systolic deviation towards the left ventricular outflow tract. Additionally, a connection between the anterior leaflet chordae tendineae and the interventricular septum, as well as narrowing of the left ventricular outflow tract with a minimum diameter of 3.2 mm, indicated left ventricular outflow tract obstruction. Mild regurgitation of the anterior leaflet of the mitral valve and mild tricuspid regurgitation were also observed. She was considered to have HCM and received oral Metoprolol as a treatment for HCM at our hospital. Regular follow-up examinations had been conducted, during which the patient exhibited slightly delayed language development, with limited ability to pronounce single words, and she did not need intervention treatment. But there were no delays in growth or motor development. When she grew up to three years old, her hypertrophy myocardial symptoms were aggravated and she had to be hospitalized for surgery.

**Figure 1.**
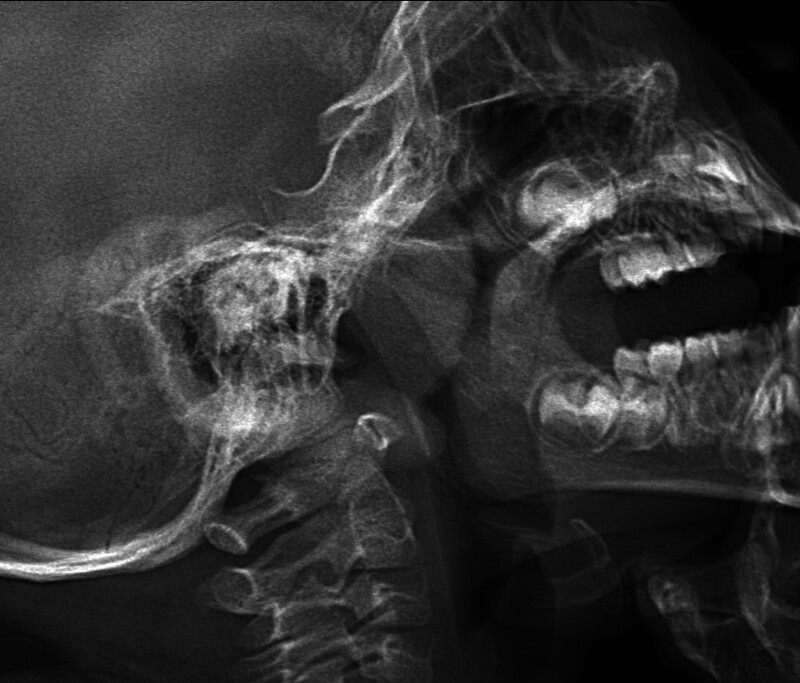
Neck X-ray: hypertrophy of the tonsils and adenoids.

Physical examination after hospitalization revealed body weight 16 kg, a pulse rate of 120 beats/min, respiratory rate of 28 breaths/min, blood pressure of 95/47 mm Hg, body temperature of 37.3 °C, and a height of 96 cm. The patient appeared alert and responsive, with thick eyebrows, large eyes, narrow palpebral fissures, proptosis, widened nostrils, protruding upper lip, slight retrusion of the mandible, red throat, and hypertrophy of the tonsils and adenoids (Fig. 1). The patient had a supple neck, exhibited smooth breathing with clear lung sounds, and no rales or wheezing were detected. Regular rhythm, moderate heart sounds, and a grade 3/6 systolic murmur at the left sternal edge between the 2nd and 3rd ribs were observed.

**Figure 2.**
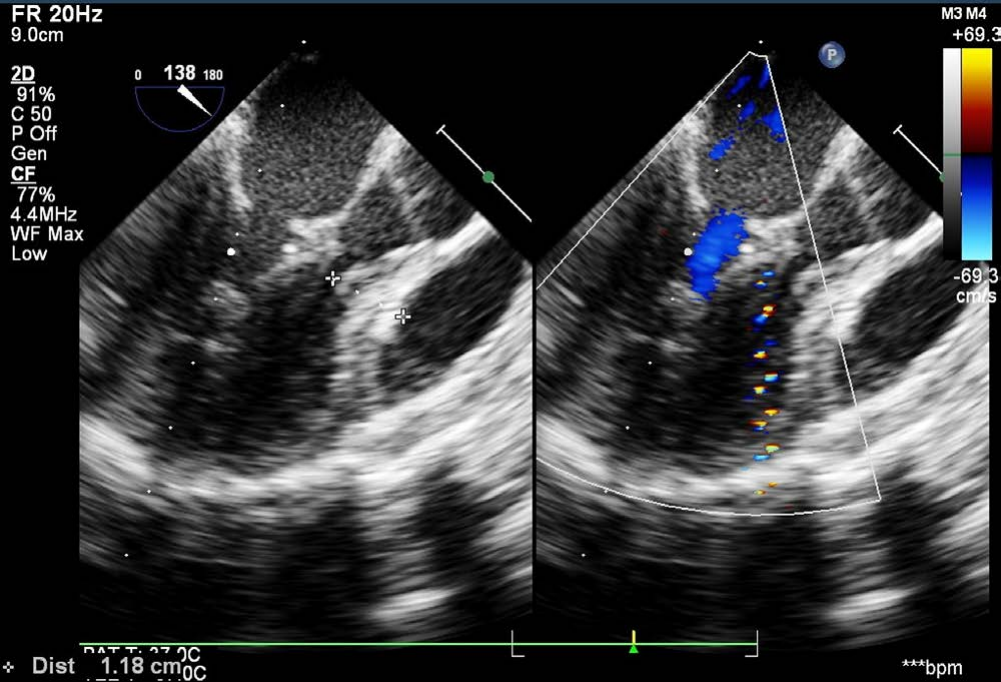
Cardiac ultrasound before surgery.

Auxiliary examinations revealed findings from The echocardiogram (June 13, 2023). The results indicated thickening of the left ventricular wall (13.1 mm), interventricular septal thickening (approximately 10.7 mm), posterior wall thickening (approximately 10.7 mm), thickening and enhancement of the anterior leaflet of the mitral valve (approximately 4.2 mm), posterior leaflet thickening (approximately 3.6 mm), thickening of the chordae tendineae (approximately 4.3 mm), hypertrophy and enhancement of the papillary muscles, resulting in the tilting of the anterior leaflet of the mitral valve towards the left ventricular outflow tract. This caused a cleft-like appearance of the left ventricular outflow tract (SAM phenomenon), and visible connection of the anterior leaflet of the mitral valve to the interventricular septum. The ejection fraction was 0.69%, and the left ventricular diastolic dimension measured 21mm (reference range: 26.4–35.2) (Fig. 2). Genetic testing analysis (Beijing Mygenostics Co., Ltd.): The tested sample showed a heterozygous mutation in the NAA10 gene, with a substitution of cytosine (C) for thymine (T) at nucleotide position 247 (c.247>T), resulting in the substitution of arginine for cysteine at amino acid position 83 (p.R83C). No variant was found at this locus in the father’s sample, and no variant was found in the mother’s sample. This mutation is considered a spontaneous mutation. Preliminary assessment indicates that this variant is pathogenic. Bioinformatics protein function prediction software REVEL predicts a potentially harmful effect, while the predictions from SIFT, PolyPhen_2, MutationTaster, and GERP+ are harmful, benign, harmful, and harmful, respectively.

Clinical diagnosis included Ogden syndrome, congenital heart disease (obstructive hypertrophic cardiomyopathy, left ventricular outflow tract obstruction, mitral valve disease, tricuspid valve regurgitation), tonsillar and adenoidal hypertrophy, and speech and language delay.

**Figure 3.**
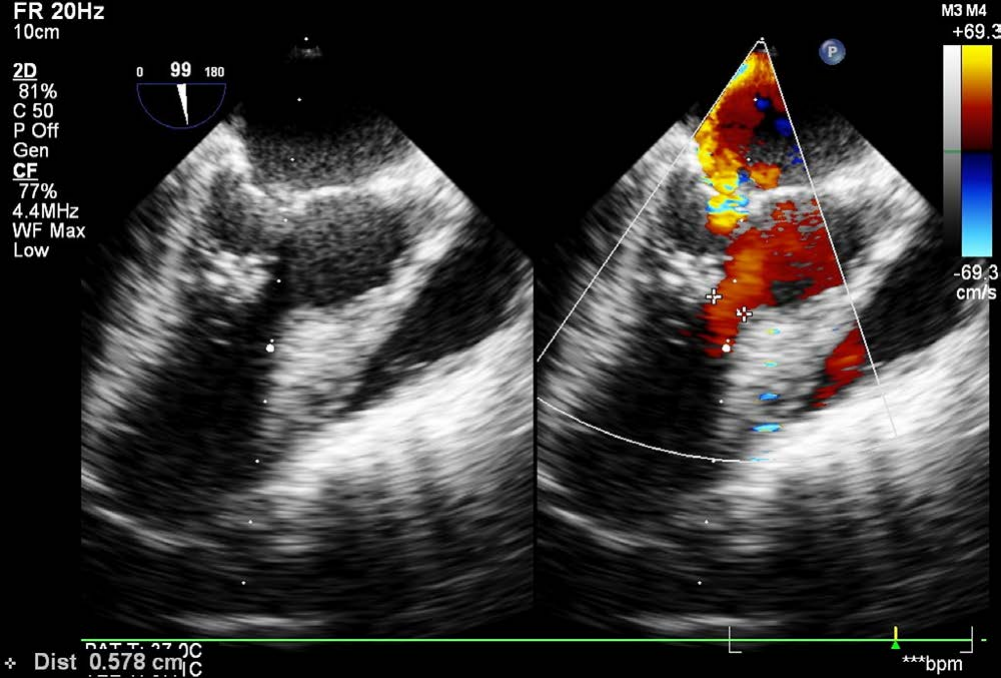
Cardiac ultrasound after surgery.

**Figure 4.**
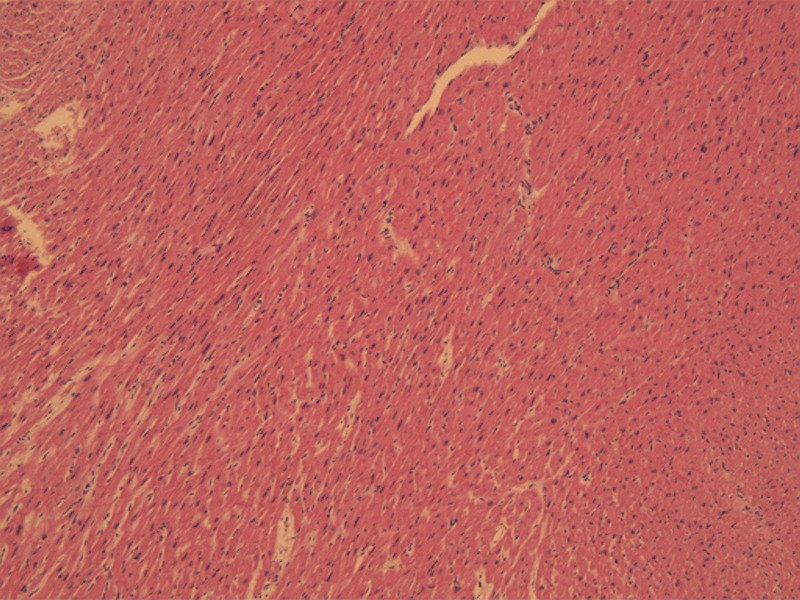
Pathology of myocardial cells: hypertrophy of fibrous and myocardial tissues.

Surgical treatment involved preoperative esophageal ultrasonography, which revealed left ventricular outflow tract hypertrophy and a significant SAM phenomenon. Abnormal tissue was observed beneath the mitral and aortic valves, and mild mitral valve regurgitation was noted. The peak flow velocity in the left ventricular outflow tract was 2.6 m/s. During cardiopulmonary bypass, the hypertrophied muscle was sutured with a bovine pericardial patch. Scar tissue and muscle of the interventricular septum, measuring approximately 1.5*1.0*0.6 cm, were removed using a blade until the base of the anterior papillary muscle was exposed. Adherent chordae tendineae beneath the anterior leaflet were excised. At the end of the surgery, esophageal ultrasonography showed the disappearance of the SAM phenomenon, clear passage in the left ventricular outflow tract, and a peak flow velocity of 1.4 m/s. The mobility of the mitral valve increased, resulting in mild regurgitation (Fig. 3). The duration of cardiopulmonary bypass was 93 minutes, the aortic cross-clamp time was 64 minutes, and the cardiac arrest time was 64 minutes. Following the surgery, the patient was transferred back to the Intensive Care Unit. On the tenth day postoperatively, the patient was discharged from the hospital. Throughout the hospitalization period, no pulmonary infections or other complications were observed. The patient’s family expressed satisfaction with the treatment process. Post-discharge, the patient still requires oral administration of metoprolol and regular follow-up examinations. The postoperative pathology report revealed hypertrophy of fibrous and myocardial tissues (Fig. 4).

The child underwent a modified Morrow procedure under cardiopulmonary bypass and experienced a favorable postoperative recovery. No pulmonary infections or significant complications were observed during this period. The patient’s family expressed satisfaction with the treatment process. After a follow-up of 1 month, no abnormalities were described in the cardiac ultrasound and main symptoms, and there was no thickening of the ventricles. However, the child will need to undergo regular follow-up examinations.

## 3. Discussion

Ogden syndrome is a rare disorder characterized by enzyme deficiency, resulting in postnatal growth retardation, severe developmental delay, and physiological defects. The incidence of this syndrome is typically less than one in a million.^[1,2,4]^ The clinical manifestations of Ogden syndrome are wide-ranging and encompass various organ systems. In this study, many of the main features observed in children diagnosed with Ogden syndrome were also present in the reported cases.^[5,6,1]^ Notably, a 14-year-old girl with the same genetic mutation described by Mandeep Sidhu et al was identified as the oldest individual with Ogden syndrome. She exhibited neurological symptoms such as autism spectrum disorder, epilepsy, extrapyramidal signs, excessive daytime sleepiness with narcolepsy, and hypertension with left ventricular hypertrophy.^[6]^ However, in her case, the cardiac problems were overshadowed by the focus on neurological and developmental issues. This oversight may contribute to the high mortality rate associated with Ogden syndrome, mainly due to arrhythmias resulting from left ventricular outflow obstruction.^[7]^

HCM is a genetic heart disease characterized by diffuse myocardial hypertrophy, typically involving the interventricular septum and anterior outer wall, with an estimated prevalence of 1 in 500 in the general population.^[8,9]^ Left ventricular outflow tract obstruction, resulting from contact between the mitral valve leaflets and the interventricular septum during ventricular ejection, is observed in a significant number of patients with HCM.^[10–13]^ Left ventricular outflow tract obstruction can lead to disabling symptoms and increase the risk of atrial and ventricular arrhythmias.^[7,14]^ Ventricular fibrillation, caused by a high left ventricular outflow tract gradient leading to coronary ischemia, is the most common cause of sudden cardiac death in HCM. Drug therapy with beta-blockers and non-dihydropyridine calcium channel blockers is the primary approach for reducing heart rate and mitigating valvular obstruction.^[15,16]^ However, in cases where medical management is inadequate or severe heart failure is present, surgical intervention may be necessary.

In this case, the patient exhibited a poor response to oral beta-blockers, with progressive thickening of the interventricular septum and ventricular wall, and a valvular gradient exceeding 50 mm Hg.^[15]^ Removing hypertrophic myocardium, which involves enlarging the cross-sectional area of the outflow tract to relieve mechanical aortic obstruction and normalize left ventricular pressure, is the recommended treatment option for relieving left ventricular outflow obstruction.^[17]^ Surgery has shown significant advantages in providing direct observation of the complex left ventricular morphology and addressing the primary mechanism leading to the aortic gradient. It reliably reverses heart failure symptoms by eliminating mechanical outflow impedance and mitral regurgitation, leading to normalization of left ventricular pressures and preserved systolic function.^[11,17]^ However, it should be noted that a small percentage of patients may continue to experience functional limitations despite successful relief of the gradient.^[17]^ Postoperative arrhythmias, such as ventricular fibrillation and atrioventricular block, may occur, but these can be managed through the implantation of pacemaker leads.

This study has certain limitations. The child needs to be followed up for a long time after surgery.

## 4. Conclusions

Once a child is diagnosed with Ogden syndrome, in addition to focusing on the nervous and growth development systems, attention should also be given to cardiac issues. If HCM is found to be concurrent, regular follow-up echocardiograms should be conducted. Early pharmacological intervention is recommended, and children with surgical indications should undergo surgery as soon as possible to reduce the risk of mortality.

## Acknowledgments

Thank you to Dr Fu from the ultrasound department and Dr Ding from the radiology department for their assistance.

## Author contributions

**Conceptualization:** Yazhou Li, Jian Tang.

**Data curation:** Feihong Li, Wenyang Wang, Zhirui Zhu.

**Formal analysis:** Yaoqin Hu.

**Resources:** Xiwang Liu.

**Writing – original draft:** Feihong Li, Wenyang Wang, Yazhou Li, Xiwang Liu, Zhirui Zhu, Jian Tang, Yaoqin Hu.

**Writing – review & editing:** Feihong Li, Wenyang Wang, Yazhou Li, Yaoqin Hu.
